# Supplementary material for: From little girls to adult women: Changes in age at marriage in Scheduled Castes from Madhya Pradesh and Uttar Pradesh, India
Source: PLoS One. 2023 Feb 13;18(2):e0281506. doi: 10.1371/journal.pone.0281506 (PMC9925072; doi:10.1371/journal.pone.0281506)
Supplement: Attachment — Submitted filename: Response to the Reviewers.docx [file pone.0281506.s001.docx]

PONE-D-22-19685

From little girls to adult women: Changes in age at marriage in Scheduled Castes from Madhya Pradesh and Uttar Pradesh, India

PLOS ONE.

**Journal Requirements:**

Dear Editor,

We are thankful for prompt processing the manuscript PONE-D-22-19685 entitled “From little girls to adult women: Changes in age at marriage in Scheduled Castes from Madhya Pradesh and Uttar Pradesh, India”. Here is point wise response to the comments:

When submitting your revision, we need you to address these additional requirements.

1. Please ensure that your manuscript meets PLOS ONE's style requirements, including those for file naming. The PLOS ONE style templates can be found at

Response:

We have made changes in the Manuscript as per suggested PLOS ONE style templates.

2. Please provide additional details regarding participant consent. In the ethics statement in the Methods and online submission information, please ensure that you have specified what type you obtained (for instance, written or verbal, and if verbal, how it was documented and witnessed). If your study included minors, state whether you obtained consent from parents or guardians. If the need for consent was waived by the ethics committee, please include this information.

Response:

As suggested, the additional details regarding participant consent were provided under a subheading ‘Statement of Ethics’ in the Methods. Please refer to the main body of the text (pp. 8-9; lines: 178-192).

3. Thank you for stating in your Funding Statement:

“RKG and GL are grateful to the Ministry of Human Resources of the Government of India, Delhi for financial support (No. 174046N04) under the Global Initiative of Academic Network (GIAN). JJ expresses her gratitude to University Grants Commission, New Delhi, for providing financial assistance for pursuing her Ph.D. and awarding a Rajiv Gandhi National Fellowship (No.F.16–29/2006 (SA–II)/817) and Post-doctoral Fellowship for Women (No. F.No.15–2/2012). MB was partially supported by the long-term strategic development financing of the Institute of Computer Science (Czech Republic RVO 67985807).The funders had no role in study design, data collection and analysis, decision to publish, or preparation of the manuscript.”

Please provide an amended statement that declares *all* the funding or sources of support (whether external or internal to your organization) received during this study, as detailed online in our guide for authors at <http://journals.plos.org/plosone/s/submit-now>.  Please also include the statement “There was no additional external funding received for this study.” in your updated Funding Statement.

Please include your amended Funding Statement within your cover letter. We will change the online submission form on your behalf.

Response:

As suggested, funding statement is revised please refer to page 19 (Lines 425-434).

4. Please include your full ethics statement in the ‘Methods’ section of your manuscript file. In your statement, please include the full name of the IRB or ethics committee who approved or waived your study, as well as whether or not you obtained informed written or verbal consent. If consent was waived for your study, please include this information in your statement as well.

Response:

As suggested, a sub heading ‘Statement of Ethics’ in the Methods. Please refer to the main body of the text (pp. 8-9; lines: 178-192).

5. We note that Figure 1 your submission contain [map/satellite] images which may be copyrighted. All PLOS content is published under the Creative Commons Attribution License (CC BY 4.0), which means that the manuscript, images, and Supporting Information files will be freely available online, and any third party is permitted to access, download, copy, distribute, and use these materials in any way, even commercially, with proper attribution. For these reasons, we cannot publish previously copyrighted maps or satellite images created using proprietary data, such as Google software (Google Maps, Street View, and Earth). For more information, see our copyright guidelines: <http://journals.plos.org/plosone/s/licenses-and-copyright>.

We require you to either (1) present written permission from the copyright holder to publish these figures specifically under the CC BY 4.0 license, or (2) remove the figures from your submission:

 a. You may seek permission from the original copyright holder of Figure 1 to publish the content specifically under the CC BY 4.0 license.

We recommend that you contact the original copyright holder with the Content Permission Form (<http://journals.plos.org/plosone/s/file?id=7c09/content-permission-form.pdf>) and the following text:

“I request permission for the open-access journal PLOS ONE to publish XXX under the Creative Commons Attribution License (CCAL) CC BY 4.0 (<http://creativecommons.org/licenses/by/4.0/>). Please be aware that this license allows unrestricted use and distribution, even commercially, by third parties. Please reply and provide explicit written permission to publish XXX under a CC BY license and complete the attached form.”

Please upload the completed Content Permission Form or other proof of granted permissions as an "Other" file with your submission.

In the figure caption of the copyrighted figure, please include the following text: “Reprinted from [ref] under a CC BY license, with permission from [name of publisher], original copyright [original copyright year].”

 b. If you are unable to obtain permission from the original copyright holder to publish these figures under the CC BY 4.0 license or if the copyright holder’s requirements are incompatible with the CC BY 4.0 license, please either i) remove the figure or ii) supply a replacement figure that complies with the CC BY 4.0 license. Please check copyright information on all replacement figures and update the figure caption with source information. If applicable, please specify in the figure caption text when a figure is similar but not identical to the original image and is therefore for illustrative purposes only.

The following resources for replacing copyrighted map figures may be helpful:

USGS National Map Viewer (public domain): <http://viewer.nationalmap.gov/viewer/>

The Gateway to Astronaut Photography of Earth (public domain): <http://eol.jsc.nasa.gov/sseop/clickmap/>

Maps at the CIA (public domain): <https://www.cia.gov/library/publications/the-world-factbook/index.html> and <https://www.cia.gov/library/publications/cia-maps-publications/index.html>

NASA Earth Observatory (public domain): <http://earthobservatory.nasa.gov/>

Landsat: <http://landsat.visibleearth.nasa.gov/>

USGS EROS (Earth Resources Observatory and Science (EROS) Center) (public domain): [http://eros.usgs.gov/#](http://eros.usgs.gov/)

Natural Earth (public domain): <http://www.naturalearthdata.com/>.

Response:

As suggested, the Figure 1 is deleted. Accordingly, the Figure number also changed in the manuscript.

6. Please review your reference list to ensure that it is complete and correct. If you have cited papers that have been retracted, please include the rationale for doing so in the manuscript text, or remove these references and replace them with relevant current references. Any changes to the reference list should be mentioned in the rebuttal letter that accompanies your revised manuscript. If you need to cite a retracted article, indicate the article’s retracted status in the References list and also include a citation and full reference for the retraction notice.

Response:

As suggested, the references are updated.

Additional Editor Comments (if provided):

The article is very interesting and reinforces the importance of the influence of education on social and economic outcomes related to girls and women. A topic of great relevance in the global agenda to reduce gender inequality. To improve the quality of the manuscript, it is necessary to carry out the corrections suggested by the reviewers.
Response:

We are highly grateful for high words of appreciation. It will really open a new path for us to work more sincerely and contribute for the betterment of humanity.

Reviewers' comments:

Reviewer's Responses to Questions

**Comments to the Author**

1. Is the manuscript technically sound, and do the data support the conclusions?

The manuscript must describe a technically sound piece of scientific research with data that supports the conclusions. Experiments must have been conducted rigorously, with appropriate controls, replication, and sample sizes. The conclusions must be drawn appropriately based on the data presented.

Reviewer #1: Yes

Reviewer #2: Partly

Response:

We are thankful for positive comments and appreciation. The comments of second reviewer are also addressed and further details of data and ethical issues are provided in the revised version of manuscript. Please refer to page 8-9; lines: 178-192.

2. Has the statistical analysis been performed appropriately and rigorously?

Reviewer #1: Yes

Reviewer #2: Yes

Response:

We are thankful for positive comments and appreciation.

3. Have the authors made all data underlying the findings in their manuscript fully available?

The [PLOS Data policy](https://eur01.safelinks.protection.outlook.com/?url=http%3A%2F%2Fwww.plosone.org%2Fstatic%2Fpolicies.action%23sharing&data=05%7C01%7Cgrazyna.liczbinska%40amu.edu.pl%7Caecfed31377a4b1a827808dab8ffebe6%7C73689ee1b42f4e25a5f666d1f29bc092%7C0%7C0%7C638025706840741802%7CUnknown%7CTWFpbGZsb3d8eyJWIjoiMC4wLjAwMDAiLCJQIjoiV2luMzIiLCJBTiI6Ik1haWwiLCJXVCI6Mn0%3D%7C3000%7C%7C%7C&sdata=YMxLXjHyz8xw63Shgbayv%2FS45F3R7dNwvAefisRXTQ8%3D&reserved=0) requires authors to make all data underlying the findings described in their manuscript fully available without restriction, with rare exception (please refer to the Data Availability Statement in the manuscript PDF file). The data should be provided as part of the manuscript or its supporting information, or deposited to a public repository. For example, in addition to summary statistics, the data points behind means, medians and variance measures should be available. If there are restrictions on publicly sharing data—e.g. participant privacy or use of data from a third party—those must be specified.

Reviewer #1: Yes

Reviewer #2: No

Response:

Data Availability Statement is added in the revised version of the Manuscript. Please refer to Page 20, Line 435-436.

4. Is the manuscript presented in an intelligible fashion and written in standard English?

PLOS ONE does not copyedit accepted manuscripts, so the language in submitted articles must be clear, correct, and unambiguous. Any typographical or grammatical errors should be corrected at revision, so please note any specific errors here.

Reviewer #1: Yes

Reviewer #2: Yes

Response:

Thanks to both reviewers.

5. Review Comments to the Author

Please use the space provided to explain your answers to the questions above. You may also include additional comments for the author, including concerns about dual publication, research ethics, or publication ethics. (Please upload your review as an attachment if it exceeds 20,000 characters)

**Reviewer #1:**

The article is very interesting and reinforces the importance of the influence of education on social and economic outcomes related to girls and women.

Response:

We are thankful for positive comments and appreciation.

The introduction, method, results and discussion are adequately described, however I have some suggestions:

Materials and methods

a) Describe in more detail the genealogical method of age estimation, used to validate the information obtained;

Response:

In the revised manuscript detail the genealogical method of age estimation provided. Please refer to the main body of the text pages 6-7; lines:135-152.

b) Detail the “crosscheck” described on page 6, line 140

now line 149 – I have marked with green font!

Response: .

In the revised manuscript detail of “crosscheck” is further illustrated please refer to Page 7, Line 161-165.

Results:
a) I suggest that the legends of the figures are next to them (Fig. 2, Fig. 3, Fig. 4).

Response:

As suggested by the Reviewer, we revised the figures and now legends are next to them.

b) Review the description in Table 2, as the data in the text is different from the data presented in the Table (Line 229-231, page 10).

Response:

In the revised Manuscript it is taken care that description of Table 2 and text is similar now. Please refer to page 12, Line 267-270.

**Reviewer #2:**

The theme presented is current and relevant. Girls' vulnerabilities and penalties for maternity and marriage are exacerbated in contexts of poverty and social inequality. The article must be published, but with an adjustment in relation to the presentation of the database. The text is very well written and brings important results on gender inequalities in the private sphere. The work innovated by resorting to cohort analysis. This work complements the analyses done for both India and looks at the positive impacts of education as a protective factor for child marriage.

Response:

We are thankful for words of appreciation; it is really inspiring for us.

The work presented is relevant to the literature and brings consistent results. Although the authors have made an effort to choose and apply the data analysis model, as well as to present and discuss the results, the same effort is not observed in the description of the database.

Response:

In the revised Manuscript, we tried to address this suggestion of the Reviewer.

It would be interesting to specify more about the data source used. What are the criteria for choosing respondents and how can these criteria bias the sample? How does this sample resemble the female population of the studied locations? To what extent can the survey results, based on this sample, be generalized to the female population of the two states?

Response:

The Manuscript is revised and a paragraph is added to describe about the data source used, criteria for choosing respondents, how does this sample resemble the female population of the studied locations, etc. Please refer to page 6 Line 119-134.

I could not identify, from the information provided, the place where the database could be consulted.

Response:

In the revised Manuscript, the district from where the data was collected is mentioned please refer to Page 6 Line 132-134.

Small suggestions:
i) It would be interesting to indicate N in the regression model tables.

Response:

As suggested, in the revised manuscript the sample size (N) is indicated in the Tables (see: Table 1; p. 11 and Table 2; p. 12.

ii) The data presented in lines 293 to 309 would be easier to understand if they were presented in tables.

Response:

As suggested, in the revised Manuscript data has been presented in Tables (see additional Table 3, p. 15 and Table 4, p.16.
